# Circulating Antibodies to Skin Bacteria Detected by Serological Lateral Flow Immunoassays Differentially Correlated With Bacterial Abundance

**DOI:** 10.3389/fmicb.2021.709562

**Published:** 2021-11-10

**Authors:** Ryan Yuki Huang, Chuen Neng Lee, Shabbir Moochhala

**Affiliations:** ^1^Canyon Crest Academy, San Diego, CA, United States; ^2^Department of Surgery, National University of Singapore, Singapore, Singapore

**Keywords:** acne vulgaris, antibody, *C. acnes*, *C. aurimucosum*, lateral flow immunoassays, *S. aureus*

## Abstract

The serological lateral flow immunoassay (LFIA) was used to detect circulating antibodies to skin bacteria. Next-generation sequencing analysis of the skin microbiome revealed a high relative abundance of *Cutibacterium acnes* but low abundance of *Staphylococcus aureus* and *Corynebacterium aurimucosum* on human facial samples. Yet, results from both LFIA and antibody titer quantification in 96-well microplates illustrated antibody titers that were not correspondent, and instead negatively correlated, to their respective abundance with human blood containing higher concentrations of antibodies to both *S. aureus* and *C. aurimucosum* than *C. acnes*. Acne vulgaris develops several unique microbial and cellular features, but its correlation with circulating antibodies to bacteria in the pilosebaceous unit remains unknown. Results here revealed that antibodies to *C. acnes* and *S. aureus* were approximately 3-fold higher and 1.5-fold lower, respectively, in acne patients than in healthy subjects. Although the results can be further validated by larger sample sizes, the proof-of-concept study demonstrates a newfound discrepancy between the abundance of skin bacteria and amounts of their corresponding antibodies. And in light of acne-correlated amplified titers of specific anticommensal antibodies, we highlight that profiling these antibodies in the pilosebaceous unit by LFIAs may provide a unique signature for monitoring acne vulgaris.

## Introduction

Human blood contains not only antibodies provoked by infections and vaccinations but also antibodies acquired by exposure to commensal bacteria (Zhao and Elson, 2018). While the anticommensal antibody repertoire has not been profiled in full, immunoglobulin G (IgG) in human blood with broad specificity to bacteria in the gastrointestinal tract has been detected (Castro-Dopico et al., 2019). These anticommensal antibodies have been documented to elicit proinflammatory activities by activation of fragment crystallizable (Fc) receptors on macrophages (Castro-Dopico et al., 2019). The serological lateral flow immunoassays (LFIAs) have been frequently used to detect the circulating antibodies (Banerjee and Jaiswal, 2018), mainly generated by infections or vaccinations including coronavirus disease 2019 (COVID-19) vaccines against SARS-CoV-2 (severe acute respiratory syndrome coronavirus 2) (Ravi et al., 2020). However, LFIA has been widely used in detection of anticommensal antibodies in people with health or disease condition.

Antibodies to *Cutibacterium acnes*, a bacterium associated with pathogenesis of acne vulgaris, in human blood have been clearly detected (Wang et al., 2018). The human sebaceous pilosebaceous unit is commonly referred to as “the seat of acne vulgaris” and harbors a heterogeneous community of microorganisms, including *C. acnes*, *Staphylococcus* spp., and *Corynebacterium* spp. (Grice and Segre, 2011). For example, *Corynebacterium aurimucosum* is often detected in sebaceous sites in humans (Blaskovich et al., 2019) and erythromycin-resistant *Staphylococcus aureus* in the comedones of acne vulgaris patients (Sitohang et al., 2019). While the pathophysiology of acne vulgaris remains unclear, many of the aforementioned microbial species have been widely associated with acne vulgaris. Features of acne vulgaris can be irregular patterns of composition and abundance of bacteria, dominance of virulent bacterial subtypes, or genetic elements or metabolites of bacteria and host cell responses, including activation of receptors and secretion of antimicrobial peptides or inflammatory cytokines in the microbiome of acne vulgaris compared with healthy skin (Chen et al., 2021).

Microbial composition results from the next-generation sequencing (NGS) platform for 16S ribosomal RNA (rRNA) gene analyses have revealed severe alterations in bacterial abundances, or dysbiotic microbiomes, in acne vulgaris. In comparing microbial profiles of healthy and acne patients, the relative abundance of *C. acnes* was similar in healthy skin compared to acne lesions, but a higher abundance of *Cutibacterium granulosum* was found in healthy skin (Rajiv et al., 2013). *C. acnes* has been classified into phylogenetic clades IA-1, IA-2, IB-1, IB-2, IB-3, IC, II, and III (Fitz-Gibbon et al., 2013). Strains from clades IA-2 (mostly ribotypes 4 and 5), IB-1 (ribotype 8), and IC (ribotype 5) are closely associated with acne vulgaris, whereas clade II strains that include ribotypes 2 and 6 are often detected in healthy skin (Lee et al., 2019). Profiles of genetic elements and metabolites of bacteria in healthy skin and acne lesions are also different. The gene-encoding Christie–Atkinson–Munch–Peterson (CAMP) factors *camp1*, *camp2*, and *camp4*, which contribute to hemolysis and inflammation, were found to be more abundantly expressed in acne lesions (O’Neill and Gallo, 2018). The *gehA* gene (PPA2105) encoding *C. acnes* lipase can increase sebum concentrations of free palmitate, which plays an important role in lipotoxic inflammasome activation of macrophages (Legrand-Poels et al., 2014). The secretion of interleukin 1α (IL-1α) via activation of Toll-like receptor 2 (TLR2) by ligands present on *C. acnes* can be detected in inflammatory acne vulgaris (Graham et al., 2004). It has been reported that the mRNA and protein levels of both IL-1β and IL-8 in acne lesions were higher than those in healthy skin (Wang et al., 2018). Here, we take advantage of LFIAs to detect the antibodies to skin bacteria including *C. acnes*, *S. aureus*, and *C. aurimucosum* in the sera of healthy subjects and acne patients. Unlike the traditional use of LFIAs for detection of infection- and vaccination-generated antibodies, we highlight that the antibodies to skin commensal bacteria on LFIAs can be an indicator for inflammatory diseases such as acne vulgaris.

## Materials and Methods

### Ethics Statement

Human blood and skin swabs were obtained from Dr. Chun-Ming Huang with an approved protocol (no. 121230) (Wang et al., 2018) by the institutional review board at University of California, San Diego (UCSD).

### Skin Swabs and DNA Extraction

Total DNA was extracted from swab samples of cheeks of 15 (eight males and seven females) individuals between the ages of 21 and 35 years without acne vulgaris on facial skin using a QIAamp DNA Mini Kit (Qiagen, Hilden, Germany) for polymerase chain reaction (PCR) (Wang et al., 2018). All individuals were asked without washing faces with water or other detergents at least 6 h before swabbing. Skin swabbing was conducted according to a method as described in previous studies (Ogai et al., 2018) with minor modifications. Concisely, a 4 × 4-cm^2^ area medial to the zygomatic prominence on bilateral cheeks was gently swabbed with a sterile cotton swab presoaked in a solution containing 0.9% sodium chloride and 0.1% Tween-20. For DNA extraction, the heads of all collected swab cottons were cut off and stored in sterile 1.5-mL centrifugation tubes at −80°C until DNA extraction. The swab heads were incubated in an enzyme solution (0.5 mL) [20 mg/mL lysozyme and 200 μg/mL lysostaphin (Sigma, St. Louis, MO, United States), in 20 mM Tris-HCl (pH 8.0), 2 mM ethylenediaminetetraacetic acid, and 1% Triton-X 100] at 37°C for 30 min. Proteinase K (20 μL) was subsequently added into the solution for 30 min, followed by deactivation of all enzymes at 95°C for 15 min.

### Next-Generation Sequencing Analysis

NGS was performed according to a protocol previously published (Godlewska et al., 2020). Briefly, PCR amplicons were sequenced utilizing Roche 454 FLX titanium instruments (Roche, Indianapolis, IN, United States). The 16S rRNA gene V4 variable region PCR primers 515/806 were selected in a single-step 30-cycle PCR. Sequences were denoised; 16S rRNA sequences were clustered into operational taxonomic units (OTUs). Final OTUs were taxonomically classified using the nucleotide basic local alignment search tool (BLASTn) against a curated database derived from GreenGenes, Ribosomal Database Project II and the National Center for Biotechnology Information. Bacteria with relative abundances ≥1% in skin of at least one individual or bacteria identified at the species level were presented.

### Preparation and Use of Human Sera

Blood was collected from healthy subjects with no prior history of acne vulgaris and acne patients between the ages of 21 and 50 years. Acne patients with multiple acne lesions (more than five lesions on the face or back skin) with different levels of severity were selected for blood collection. Acne patients were selected from participants who did not receive topical corticosteroids or systemic isotretinoin, immunosuppressives, chemotherapeutic agents, anti-inflammatory biologic, or oral antimicrobial agents within 30 days prior to the blood collection. Individual sera from each patient, not pooled sera from multiple patients, were used for experiments in this study.

### Lateral Flow Immunoassay Fabrication of Antibody Detection

The LFIAs with test and control zones were fabricated using three pads (sample, conjugate, and absorbent pads) and one nitrocellulose (NC) membrane (Membrane Technologies Inc., Harrisburg, PA, United States). HAuCl_4_ (1 mM) (Sigma) was boiled at 200°C before addition of trisodium citrate dihydrate (1%). The gold nanoparticles (AuNPs) were conjugated with protein A of *S. aureus* (SPA) (20 μg) (Sigma) in 1 mL borax buffer containing boric acid and sodium borax, pH 5–6. One percent bovine serum albumin (BSA) in phosphate-buffered saline (PBS) was used for blocking. The SPA-conjugated AuNPs (100 μL) was pipetted onto the conjugate pad. Lysates of bacteria [*C*. *acnes* (ATCC 6919 and a skin isolate), *C. aurimucosum* (ATCC 700975), and *S. aureus* 113 (ATCC 35556)] were prepared by heat at 100°C for 1 h. Bacterial lysates and goat anti–human IgG (H+L) (3 μg) (Thermo Fisher Scientific, Waltham, MA, United States) were placed onto test and control zones on an NC membrane, respectively. The 5% sera (200 μL) were added onto the sample pad to start reaction. Antibodies in sera were captured by SPA-conjugated AuNPs in a conjugate pad and then flowed through to an NC membrane. The purple spots were formed when antibodies interact with bacterial lysates or anti–mouse IgG (H+L) on NC membranes. The intensities of purple spots on test zones relative to those on control zones were quantified by ImageJ software 1.50 b (National Institutes of Health, Bethesda, MD, United States).

### Quantification of Antibody Titers

Bacterial lysates (0.1 μg/well) or BSA diluted in 50 μL PBS was coated onto a 96-well microplate for 4°C for 24 h. After blocking in PBS with 1% BSA, diluted sera were added to the wells and incubated for 2 h. A goat anti–human IgG (H+L) IgG–horseradish peroxidase (HRP) conjugate (Thermo Fisher Scientific) (1:10,000 dilution) was added and incubated for 1 h. HRP activity was measured by an OptEIA^®^ Reagent Set (Thermo Fisher Scientific). The optical density (OD) of each well was measured at 450 nm subtracted from 570 nm (OD_57__0__–__4__50_). The endpoint was defined as the four-time dilution of sera producing the same OD_57__0__–__4__50_ as a 1/100 dilution of sera added to the BSA-coated wells. Sera negative at the lowest dilution tested were assigned endpoint titers of 100. The data were presented as geometric mean endpoint serum titers.

### Quantitative Reverse Transcription–Polymerase Chain Reaction

To determine the mRNA expression level of *C. acnes* lipase (WP_002530746.1), total cellular RNA was extracted from *C. acnes* using the RNeasy Mini Kit (Thermo Fisher Scientific). The RNA was reverse transcribed to cDNA using an iScript cDNA synthesis kit (Bio-Rad, Hercules, CA, United States) and amplified by reverse transcription (RT)–qPCR on the CFX96 real-time system (Bio-Rad) using forward (TCACTGATGAAGATCAACGCAC) and reverse (TGCGAATGTCCGACGAAGTCGA) primers. The comparative delta–delta cycle threshold (ΔΔCT) was used to quantify the gene expression, which was normalized to the expression level of 16S rRNA of *C. acnes*.

### Statistical Analysis

Statistical analysis was performed by unpaired *t*-test using GraphPad Prism software. *p* < 0.05 (*), *p* < 0.01 (**), and *p* < 0.001 (***) were considered significant. The mean ± standard deviation (SD) for at least three independent experiments was calculated.

## Results

### High-Abundance *C. acnes* and Low-Abundance *S. aureus* and *C. aurimucosum* in the Skin Microbiome

**FIGURE 1:**
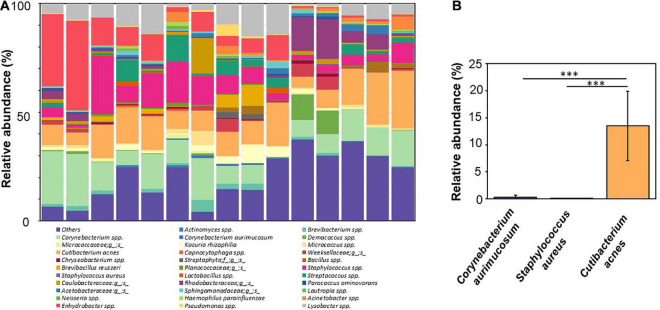
NGS-analyzed microbiome composition at species level with relatively high abundance of *C. acnes* and low abundance of *S. aureus* and *C. aurimucosum* in the human skin. **(A)** NGS analyzed by a Roche 454 FLX titanium instrument using 16S rRNA gene was conducted after extraction of DNA from swab samples of facial skin of 15 human subjects. Four bacteria species [*Corynebacterium* spp. (pale green), *C. acnes* (orange), *Staphylococcus* spp. (pink), and *Enhydrobacter* spp. (pink–red)] with average relative abundances ≥10% in more than 5 of 15 human subjects were detected. Bacteria with relative abundances ≥1% in skin of at least one individual or bacteria identified at the species level were presented. Bacteria with an abundance < 1% were pooled and indicated as “Others.” **(B)** The relative abundances of *C. acnes* (orange), *S. aureus* (purple), and *C. aurimucosum* (blue) are displayed. The mean ± SD obtained from 15 subjects was calculated. *p* < 0.001 (***), two-tailed *t*-test, is shown.

NGS analysis was performed using skin swab samples of facial skin collected from 15 young adults aged 21–35 years. The relative abundance of facial bacteria at the species level was determined by detecting 16S rRNA expression. Seven bacteria species with relative abundances greater than 0.02% in more than five skin swab samples were identified. These seven bacteria were *C. acnes*, *S. aureus*, *C. aurimucosum*, *Kocuria rhizophila*, *Brevibacillus reuszeri*, *Haemophilus parainfluenzae*, and *Paracoccus aminovorans*. The taxonomical assignment and relative abundance estimates for detected OTUs identified 32 groups of bacteria, which were several different unknown species under known genus or family (Figure 1A). Four bacteria species including *C. acnes*, *Corynebacterium* spp., *Staphylococcus* spp., and *Enhydrobacter* spp. with average relative abundances ≥10% in more than 5 of 15 human subjects were detected. Besides four species above, other bacteria with relative abundances ≥10% found on the skin of at least two human subjects included *Dermacoccus* spp., *Lysobacter* spp., *Streptococcus* spp., and bacteria in the Rhodobacteraceae family. Among the identified bacterial species, *C. acnes*, *S. aureus*, and *C. aurimucosum* with a relative abundance of 13.48% ± 6.43%, 0.02% ± 0.02%, and 0.24% ± 0.41% (Figure 1B), respectively, were often found around sebaceous glands on human face. The data demonstrated that the human face contains far more *C. acnes* than *S. aureus* and *C. aurimucosum*.

### Higher Titer of Antibodies to *S. aureus* and *C. aurimucosum* Than *C. acnes* in Human Sera

**FIGURE 2:**
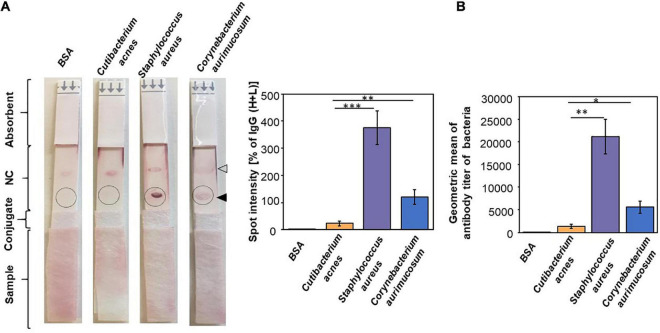
Production of a higher titer of antibodies to *S. aureus* and *C. aurimucosum* than *C. acnes* in human sera. **(A)** BSA or the lysate of *C. acnes*, *S. aureus*, or *C. aurimucosum* (3 μg) was on the test line (solid triangle) of an NC membrane of an LFIA. The anti–human IgG (H+L) was spotted on the control line (open triangle) of an NC membrane of each LFIA. After adding human sera (5%, 200 μL) onto sample pads, the relative intensities of purple spots (circles) were quantified by normalizing those of IgG (H+L) spots. **(B)** The antibodies (IgG) to *C. acnes*, *S. aureus*, or *C. aurimucosum* were detected by adding human sera to 96-well microplates coated with lysates of *C. acnes*, *S. aureus*, or *C. aurimucosum*, respectively. Quantitative antibodies were measured by using the endpoint of diluted sera producing the same OD_57__0__–__4__50_ as a 1/100 dilution of sera added to the BSA-coated wells. Individual, not pooled, sera from three human subjects were used for detection of antibodies on LFIAs and 96-well microplates. Sera used for LFIAs and 96-well microplates were from the same human subject. *p* < 0.05 (*), *p* < 0.01 (**), and *p* < 0.001 (***) with mean ± SD were obtained from three independent experiments.

It has been shown that there is a systemic humoral response against commensal bacteria in healthy individuals (Sterlin et al., 2020). To examine the correlation between the abundance of bacteria and their antibody titers in humans, the level of IgG toward the antigens of *C. acnes*, *S. aureus*, and *C. aurimucosum* was evaluated by the reactivity of the human sera with the corresponding bacterial lysates. The total lysates of *C. acnes* ATCC 6919, *S. aureus* 113 ATCC 35556, or *C. aurimucosum* ATCC 700975 were spotted on the test line of NC membranes of an LFIA. The anti–human IgG (H+L) was spotted on the control line of NC membrane of each LFIA. The relative intensities of purple spots on LFIA corresponding to antibody titers were quantified by normalizing those of IgG (H+L) spots. As shown in Figure 2A, the level of IgG against *S. aureus* or *C. aurimucosum* was approximately 16- or 5-fold, respectively, higher than that of IgG against *C. acnes*. Experiments of using bacterial lysates coated onto 96-well plates for probing with the corresponding sera were conducted to quantify the titers of antibodies to *C. acnes*, *S. aureus*, and *C. aurimucosum.* The mean titers of antibodies to *C. acnes*, *S. aureus*, and *C. aurimucosum* in 2% human sera were 1:1,333, 1:21,200, and 1:5,600, respectively (Figure 2B). Results of quantifying antibodies on 96 wells strongly confirmed that the LFIA data on the titer of antibodies to *S. aureus* or *C. aurimucosum* were much higher than those of antibodies to *C. acnes* in human sera. Taking Figures 1, 2 together, we found that (1) antibodies to *C. acnes*, *S. aureus*, or *C. aurimucosum* were detectable in human sera; however, (2) *S. aureus* and *C. aurimucosum* with a low bacterial abundance elicited a higher antibody titer than *C. acnes*, which was found to be highly abundant on face skin.

### High Levels of Antibodies to *C. acnes* and Low Levels of Antibodies to *S. aureus* in Acne Patients

**FIGURE 3:**
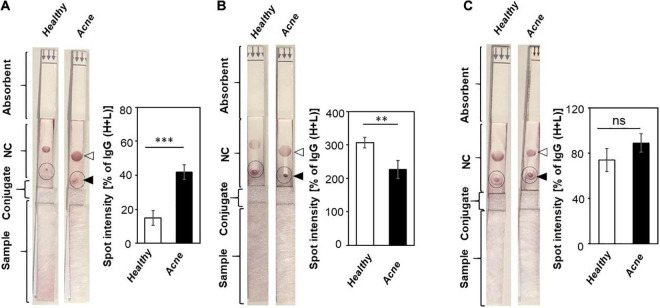
Detection of antibodies to *C. acnes*, *S. aureus*, and *C. aurimucosum* in acne patients and healthy subjects. The lysates (3 μg) of **(A)** *C. acnes* ATCC 6919, **(B)** *S. aureus* 113 (ATCC 35556), or **(C)** *C. aurimucosum* (ATCC 700975) were spotted on the test lines (solid triangle), and anti–human IgG (H+L) (open triangle) was spotted on control lines of NC membranes of LFIAs. Five percent sera (200 μL) from healthy subjects and acne patients were added into the sample pads. The high amounts of antibodies to *C. acnes* ATCC 6919 and low amounts of antibodies to *S. aureus* were detected in sera of acne patients compared to sera of healthy subjects. The intensities of IgG (H+L) spot served as 100%. The relative intensities of purple spots (circles) corresponding to the levels of antibodies were normalized by those of IgG (H+L) spots. Individual sera from three human subjects and acne patients were used. *p* < 0.01 (**) and *p* < 0.001 (***) with mean ± SD were calculated from three healthy subjects and acne patients. ns, not significant.

Literature has revealed that the imbalance of different *C. acnes* phylotypes, together with a dysbiosis of the skin microbiome, may be a signature of acne vulgaris (Dréno et al., 2020). To determine whether the titer of antibodies to bacteria such as *C. acnes*, *S. aureus*, and *C. aurimucosum* has a correlation with the acne vulgaris, sera from acne patients and healthy subjects were pipetted on sample pads of LFIAs with NC membranes immobilized with lysates of *C. acnes*, *S. aureus*, or *C. aurimucosum*. Purple spots were formed when the binding of antibodies in the sera to the bacterial lysates occurred. As shown in Figure 3A, addition of sera of acne patients onto LFIAs immobilized with lysates of *C. acnes* exhibited a more than threefold higher spot intensity than addition of sera of healthy subjects. The data indicated that acne patients had a higher titer of antibodies to *C. acnes* than healthy subjects. Results from LFIAs immobilized with lysates of *S. aureus* (Figure 3B) displayed that sera of acne patients contained approximately 1.5-fold lower levels of antibodies to *S. aureus* than the sera of healthy subjects. However, the level of systemic IgG directed against *C. aurimucosum* on LFIAs, although it was detectable, was very similar between acne patients and healthy subjects (Figure 3C). The differential levels of antibodies to *C. acnes* and *S. aureus* in acne patients and healthy subjects may serve as a signature of acne vulgaris.

### An Elevated Titer of Antibodies to a Strain of *C. acnes* Isolated From Acne Lesions

**FIGURE 4:**
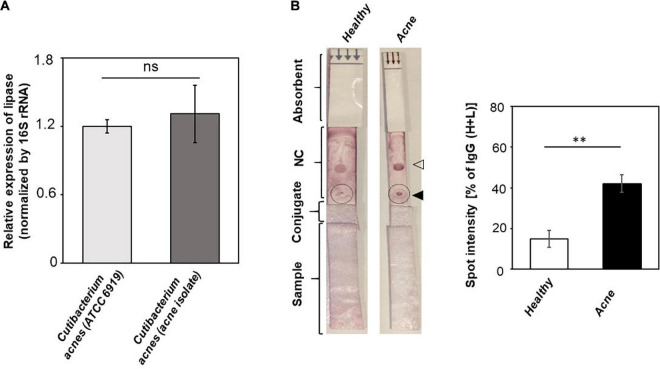
A higher level of antibodies to a *C. acnes* 74 strain isolated from acne lesions in acne patients as compared to healthy subjects. **(A)** The expression of the *gehA* gene encoding lipase in *C. acnes* ATCC 6919 and a *C. acnes* 74 strain was detected by RT-qPCR. The relative expression of the *gehA* gene was normalized to 16S rRNA. Data shown represent the mean ± SD of an experiment performed in triplicate. ns, not significant. **(B)** LFIAs were spotted with 3 μg of lysates (solid triangle) of a *C. acnes* 74 strain and anti–human IgG (H+L) (open triangle). Five percent individual sera (200 μL) from healthy subjects (*n* = 3) and acne patients (*n* = 3) were inserted into the sample pads. The relative levels of antibodies (circles) to *C. acnes* 74 strain were calculated as described in Figure 3 legend. *p* < 0.01 (**) with mean ± SD is shown.

Although antibodies in sera can react to antigens of *C*. *acnes* ATCC 6919 (Figure 2), a commonly used laboratory strain originally isolated from facial skin of an acne patient (Yu et al., 2015), we tested the recognition of circulating antibodies to a *C. acnes* 74 strain, which was previously isolated from acne biopsies (Wang et al., 2018). 16S rRNA sequence of the *C. acnes* 74 strain shared 99.35% identity to a *C. acnes* strain 4873 (Supplementary Figure 1). Results from RT-qPCR analysis demonstrated that there was no difference in mRNA level of the *gehA* gene encoding lipase between *C. acnes* ATCC 6919 and the *C. acnes* 74 strain (Figure 4A). In agreement with results in Figure 3, we found that purple spots on LFIAs pipetted with sera of acne patients were approximately threefold darker than those on LFIAs pipetted with sera of healthy subjects (Figure 4B), illustrating that acne patients carried higher amounts of antibodies to *C. acnes* 74 strain than healthy subjects.

## Discussion

*Corynebacterium* spp. is commonly present in healthy human flora. Several *Corynebacterium* spp., including C. *aurimucosum*, *Corynebacterium tuberculostearicum*, *Corynebacterium simulans*, *Corynebacterium kroppenstedtii*, and *Corynebacterium amycolatum*, are predominantly present in sebaceous sites (Byrd et al., 2018). Although *Corynebacterium* spp. are mostly innocuous, some species can cause human disease, most notably diphtheria, a condition caused by the diphtheria toxin secreted by *Corynebacterium diphtheriae* (Wenzel et al., 2020). Results from NGS analysis showed that *Corynebacterium* spp. accounted for approximately 10% of the total bacteria in human skin, and *C. aurimucosum* accounted for a fraction of that with a relative abundance of < 0.3% (Figure 1). Despite this, a high level of antibodies to *C. aurimucosum* was detected in the human sera (Figure 2). A possible explanation for the discrepancy in relative abundance and antibody titer is that other antibodies to *Corynebacterium* spp. may have cross-reacted to *C. aurimucosum* antigens in LFIAs and enzyme-linked immunosorbent assay (ELISA), supported by the detection of antibodies to *Corynebacterium parvum* (Wolberg et al., 1977), *C. diphtheriae* (Wenzel et al., 2020), or *Corynebacterium jeikeium* (Clark et al., 1990) in human blood. It is also possible that *C. aurimucosum* expresses highly antigenic proteins and, in turn, induced elevated productions of antibodies against *C. aurimucosum*.

*S. aureus* is frequently isolated from the anterior nares, which is assumed to be the main reservoir for the spread of the pathogen-associated skin diseases such as atopic dermatitis (van Mierlo et al., 2021). However, several *S. aureus* strains have been collected from pus cell of nodulocystic and pustular acne of volunteers (Khan et al., 2015). In parallel with *C. aurimucosum*, *S. aureus* has a relatively low relative abundance (0.03% ± 0.02%) (Figure 1) yet provoked tremendous antibodies in the blood (Figure 2). Superantigens including staphylococcal enterotoxins and toxic shock syndrome toxin-1 have been found in *S. aureus* (Schlievert et al., 2008). Expression of these superantigens may explain the elevated titer of antibodies to *S. aureus* in the bloodstream. As shown in Figure 3B, the levels of antibodies to *S. aureus* in acne patients were approximately 1.5-fold lower than those in healthy subjects. Previous studies have demonstrated that *S. aureus* can hijack *C. acnes* to intensify its virulence via interaction of *S. aureus* β-hemolysin with *C. acnes* CAMP factor (Lo et al., 2011). Thus, it is worth investigating whether a decrease in antibodies, especially neutralizing antibodies, to *S. aureus* augments the interaction of *S. aureus* with *C. acnes*, exaggerating the pathogenesis of acne vulgaris. While the expression of highly antigenic proteins in *S. aureus* could explain the negative correlation between relative abundance and circulating antibodies of *S. aureus*, *C. acnes* presented a negative correlation but in reverse: High abundance on facial skin provoked comparatively lower antibodies (Figures 1, 2). Although *C. acnes* may enter the dermal layer upon inflammation or follicle rupture in an acne lesion, the bacterium is an anaerobe present within the retained sebum in the pilosebaceous ducts (Kabashima et al., 2019). Thus, although *C. acnes* bacteria are abundant in the sebaceous pilosebaceous unit, the epidermal Langerhans cells, skin-resident dendritic cells, may not easily access *C. acnes* within an intact sebaceous follicle to present antigens. The relative abundance of bacteria on the skin surface of healthy subjects (Figure 1) was used for comparing its correlation with the profile of antibodies to bacteria. In fact, in comparing skin surface with hair follicles and healthy skin with acne lesions, the composition and relative abundance of bacteria in those skin microbiomes are different (Lousada et al., 2021). For example, *C. acnes* was dominant in the hair follicles of healthy skin, whereas additional species including *C. tuberculostearicum* became detected in the hair follicles of acne lesions (Bek-Thomsen et al., 2008). The shift of microbiome from healthy skin to acne lesions may affect the production of antibodies to each bacterium in skin. Although we obtained sera from healthy subjects with no prior history of acne vulgaris and acne patients with multiple acne lesions (Figure 3), these human subjects may have the history of infectious diseases that provoke the antibodies to *C. acnes*, *S. aureus*, or *C. aurimucosum.* It has been known that *C. acnes* was associated with the prosthetic joint infection (Boisrenoult, 2018). Clinical reports in skin infection with *S. aureus* (Kabashima et al., 2019) and urinary tract infection with *C. aurimucosum* (Lo et al., 2015) have been documented.

In this study, protein A from *S. aureus* was utilized to conjugate AuNPs for binding Fc regions of various immunoglobulins in mouse sera. Although protein A conjugated on the surface of AuNPs can simultaneously capture different antibodies in a single serum sample, it has been reported that protein A exhibited some antibody subclass differences in various species (Nelson and Reichert, 2009). Protein A elicits a higher affinity for human IgG (IgG1 and IgG2) than IgM and IgA and interacts strongly with IgG2a and IgG3 compared to IgG1 in mice (Rodrigo et al., 2015). However, it has been reported that colonization of commensal bacteria in humans primarily triggered the production of IgA by B cells (Keppler et al., 2021). Although the binding of protein A with subclasses of antibodies in mice is not yet determined, replacement of protein A with protein L (Rodrigo et al., 2015), a *Peptostreptococcus magnus* protein with a high affinity to representatives of most antibody classes, may be able to broadly capture different classes of antibodies including IgA in mice.

Antibodies to exocellular enzymes, cell wall fractions, or membrane-binding proteins of *C. acnes* were measurable in acne patients (Lodes et al., 2006). In some cases, there is a positive correlation between the titers of these antibodies and the severity of acne vulgaris (Ashbee et al., 1997). The high levels of IgG1 and IgG3 were detected in severe acne patients, whereas IgG2 was predominantly found in moderate and severe patients (Ashbee et al., 1997). Despite antibodies to *C. acnes* produced in acne patients (Webster et al., 1985; Ingham et al., 1987), the recurrence rate of acne vulgaris was high. In fact, acne patients were demonstrated to yield higher levels of antibodies to *C. acnes* than healthy subjects (Figures 3A, 4). It is possible that insufficient amounts of protective antibodies to *C. acnes* for prevention of acne vulgaris thereby lead to recurrence of acne vulgaris (Nakatsuji et al., 2008). Furthermore, age is a potential factor, as it has been reported that opportunistic *C. acnes* ribotypes are predominantly present in lesions of acne vulgaris in adolescents and young adults (Barnard et al., 2016), whereas the abundance of *C. acnes* declined in aged skin (Collier et al., 2008), and the abundance of *Corynebacterium* spp. conversely increased on the aged skin (Li et al., 2014). Still, a small sample size of participants aged from 21 to 50 years was used for this study, and increasing the sample size by including broader age groups (such as 0–20 years, ≥ 51 years) will be needed to accurately reflect the change in the skin microbiome of acne patients by age and its corresponding antibodies over the lifetime of humans.

The serum antibodies to commensal whole bacteria or their proteins as antigens are present in healthy human subjects. For example, IgG recognition of gut commensal antigens is detectable in childhood (Christmann et al., 2015). Although *C. acnes* is one of skin resident bacteria, it is commonly detected in the human gut (Gunathilake et al., 2019). Anti–*Escherichia coli* IgG, particularly antiflagellin antibodies, is detectable in blood (Jostins et al., 2014). Theoretically, it is unusual for the systemic adaptive immunity to be primed against the commensal bacteria under normal circumstances. It is not known how B cells produce antibodies to gut commensal bacteria, but it likely follows exposure to commensal bacteria that enter the lamina propria and beyond via disrupted intestinal barrier. Mucosal surfaces are protected specifically by secretory IgA and IgM, which are transported by the epithelial polymeric immunoglobulin receptor (Brown, 1978). It has been reported that the deficiency of epithelial polymeric immunoglobulin receptor resulted in the abrogation of IgA and IgM transcytosis, leading to an increase in serum IgG antibodies against gut commensals (Johansen et al., 1999). Although it is not clear how B cells can yield antibodies to skin commensal bacteria, it is well recognized that skin is an immune organ, and Langerhans cells can extend dendrites to sample the antigens of bacteria on the surface of skin (Guttman-Yassky et al., 2019).

Whether the production of antibodies to commensal bacteria in humans is beneficial or harmful to immunity is unknown. Neutralizing antibodies from vaccinations against *C. acnes* provide effective biologics to reduce inflammation in models of cells, mice (Wang et al., 2018), and *ex vivo* using acne biopsies (Nakatsuji et al., 2008; Wang et al., 2018). However, it has been illustrated that anticommensal IgG can trigger the NOD-, LRR-, and pyrin domain-containing protein 3 (NLRP3) inflammasome signaling to induce helper T cell 17–driven inflammation via activation of Fcγ receptors on macrophages (Castro-Dopico et al., 2019). Recent discovery unveiled that IgG variants without core fucosylation caused severe inflammation through increased FcγIIIA receptor affinity (Chakraborty et al., 2021; Larsen et al., 2021). Afucosylated IgG promoted secretion of proinflammatory cytokines in macrophages and correlated with COVID-19 severity (Larsen et al., 2021). These findings indicated that the severity of inflammatory can be regulated by heterogeneity of IgG. Although the (a)fucosylation of IgG induced by *C. acnes* was not determined, our results, through the detection of antibodies to C. *acnes* on LFIA, provide a signature to reflect the inflammatory acne vulgaris.

Microbial dysbiosis, a change in the bacterial abundance changes from normal to disease, can be used as a surrogate marker of human diseases. The 16S rRNA sequencing via NGS analysis is the most widely used method to examine the microbial dysbiosis. The NGS analysis can be time-consuming and labor-intensive and requires expensive equipment. Establishing a pattern to indicate the alternations in the levels of antibodies to each bacterium in the dysbiotic microbiome by LFIAs can be quickly achieved without specialized equipment as the results of antigen–antibody reaction on LFIAs can be visualized in 15–20 min (Figure 3). Although the detection of low concentrations of antibodies by LFIAs could give rise to false-negative or false-positive results compared with ELISA (La Rosa Fabian and Urquizo Briceno, 2020), many techniques have been developed to improve the sensitivity and specificity of the diagnosis (Hu et al., 2021). Recently, deep learning–based LFIAs and multiple-antigen spotted circular flow immunoassays (CFIAs) have been developed in our group (Huang and Herr, 2021). Bacterial lysates are mixtures of antigens derived from inactivated microorganisms. Spotting bacterial lysates on LFIAs can generate results due to cross-reactions of antibodies induced by different bacteria. Immobilization of various bacteria-specific antigens on a CFIA will avoid the cross-reactions of antibodies in a blood sample and simultaneously detect multiple antigen-antibody reactions in one assay. Despite that deep learning–based LFIAs or CFIAs can establish a calibration curve for antibody quantification, a cutoff value to eliminate the source of cross-reactions and background is needed (Zhang et al., 2020). When the improvements are accomplished, a signature of antibodies to different bacteria on LFIAs or CFIAs may allow physicians and medical staff to achieve diagnostic results within minutes in the future.

## Data Availability Statement

The datasets presented in this study can be found in online repositories. The names of the repository/repositories and accession number(s) can be found in the article/Supplementary Material. And I detected the following words and expressions: Global list: 16s rRNA, 16s ribosomal.

## Ethics Statement

An approved protocol (no. 121230) (Wang et al., 2018) by the institutional review board at University of California, San Diego (UCSD). The patients/participants provided their written informed consent to participate in this study.

## Author Contributions

RH wrote manuscript and performed experiments. SM and CL designed experiments and edited manuscript, respectively. All authors contributed to the article and approved the submitted version.

## Conflict of Interest

The authors declare that the research was conducted in the absence of any commercial or financial relationships that could be construed as a potential conflict of interest.

## Publisher’s Note

All claims expressed in this article are solely those of the authors and do not necessarily represent those of their affiliated organizations, or those of the publisher, the editors and the reviewers. Any product that may be evaluated in this article, or claim that may be made by its manufacturer, is not guaranteed or endorsed by the publisher.
