# Supplementary material for: Circulating Antibodies to Skin Bacteria Detected by Serological Lateral Flow Immunoassays Differentially Correlated With Bacterial Abundance
Source: Front Microbiol. 2021 Nov 10;12:709562. doi: 10.3389/fmicb.2021.709562 (PMC8635989; doi:10.3389/fmicb.2021.709562)
Supplement: Supplementary file 1 [file Presentation_1.pdf]

Supplementary material

Circulating Antibodies to Skin Bacteria on Serological Lateral Flow Immunoassays as Signatures of Acne Vulgaris

Ryan Yuki Huang [1], Chuen Neng Lee[2] and Shabbir Moochhala [2,*]

[1]Canyon Crest Academy, San Diego, CA, USA

[2]Department of Surgery, National University of Singapore, Singapore

***Correspondence:** A/P Shabbir Moochhala (Ph. D), Email: phcsmm@nus.edu.sg, Department of Surgery, Yong Loo Lin School of Medicine, National University of Singapore, 1E, Kent Ridge Wing, Singapore 119228.

Phone No: +65 67725249, Fax No: +65 67778427

Figure legend

FIGURE S1 I 16S rRNA sequence of a human skin isolate of *C. acnes* 74 strain using the 16S rRNA 41 forward (F) 5’-GAG TTT GAT CCT GGC TCA-3’ and reverse (R) 5’-ACG GCT AAC TTG TTA CGA CT-3’ 42 primers.

FIGURE S1

NNCATGCGGGTGCTACACATGCAGTCGAACGGAAAGGCCCTGCTTTTGTGGGGTG
CTCGAGTGGCGAACGGGTGAGTAACACGTGAGTAACCTGCCCTTGACTTTGGGATA
ACTTCAGGAAACTGGGGCTAATACCGGATAGGAGCTCCTGCTGCATGGTGGGGGTT
GGAAAGTTTCGGCGGTTGGGGATGGACTCGCGGCTTATCAGCTTGTTGGTGGGGT
AGTGGCTTACCAAGGCTTTGACGGGTAGCCGGCCTGAGAGGGTGACCGGCCACAT
TGGGACTGAGATACGGCCCAGACTCCTACGGGAGGCAGCAGTGGGGAATATTGCA
CAATGGGCGGAAGCCTGATGCAGCAACGCCGCGTGCGGGATGACGGCCTTCGGG
TTGTAAACCGCTTTCGCCTGTGACGAAGCGTGAGTGACGGTAATGGGTAAAGAAGC
ACCGGCTAACTACGTGCCAGAGCCCGGGTAAATA
